# Severe fever with thrombocytopenia syndrome virus from ticks: a molecular epidemiological study of a patient in the Republic of Korea

**DOI:** 10.1007/s10493-023-00783-6

**Published:** 2023-03-16

**Authors:** Seong Yoon Kim, Choong Won Seo, Hee Il Lee

**Affiliations:** 1 Division of Vectors and Parasitic Diseases, Bureau of Infectious Disease Diagnosis Control, Korea Disease Control and Prevention Agency, 187 Osongsaengmyeong 2-ro, Cheongwon-gun, Cheongju-si, Chungcheongbuk-do 363-951 Republic of Korea; 2 Department of Medical Laboratory Science, Dong-Eui Institute of Technology, 54 Yangji-ro, Busanjin-gu, Busan, 47230 Korea

**Keywords:** SFTS, SFTSV, Patient, Questing tick, *Haemaphysalis longicornis*, Republic of Korea

## Abstract

Severe fever with thrombocytopenia syndrome (SFTS) is a tick-borne infectious disease caused by *Dabie bandavirus*, commonly called SFTS virus (SFTSV). In the Republic of Korea (ROK), 1,504 cases of SFTS have been reported since the first human case was identified in 2013 until 2021. However, no case exists to provide molecular evidence between questing tick and patients with confirmed SFTS in the same living environment. In this study, we investigated the presence of ticks near the area of a patient infected with SFTSV. Ticks were collected by flagging and dry ice-baited traps at three spots in the vegetation around the patients’ residence in Chuncheon City, Gangwon Province (ROK). Among the tick samples collected, the presence of SFTSV was genetically determined using reverse transcription PCR, followed by the phylogenetic analysis of the tick virus sequences and SFTSV found in the patient. In total 1,212 *Haemaphysalis longicornis* ticks were collected, and SFTSV was detected at a minimum infection rate of 5.3% (33 pools/618 tested ticks). The sequences of SFTSV in ticks were 99.6–100% identical with the patient’s SFTSV in the M segment. To the best of our knowledge, this study is the first case to provide a molecular correlation between SFTSV in questing ticks collected from residence and patient with SFTS in the ROK. The present results provide useful information for the epidemiological investigation of patients with SFTS using ticks as vectors of SFTSV.

## Introduction

Severe fever with thrombocytopenia syndrome (SFTS) is a tick-borne zoonotic infectious disease caused by *Dabie bandavirus* (formerly SFTS virus, SFTSV) belonging to the genus *Bandavirus* in the family *Phenuiviridae* (Walker et al. 2021). After the first case was discovered in China, in 2009 (Yu et al. 2011), SFTS cases were subsequently reported in Japan (Takahashi et al. 2014), the Republic of Korea (ROK) (Kim et al. 2013), Taiwan (Republic of China) (Lin et al. 2020), and Vietnam (Tran et al. 2019). This disease presents with different clinical symptoms, including high fever, physical exhaustion, diarrhea, thrombocytopenia, and leukopenia (Yu et al. 2011). Since the first case of SFTS was reported in the ROK in 2013, the number of patients with SFTS is increasing annually, totaling 1,504 cases with a fatality rate of 18.4% (277 deaths) until 2021 in the ROK (Korea Disease Control and Prevention Agency 2022).

Although previous studies reported human-to-human transmission (Chen et al. 2013) and active handling (Yamanaka et al. 2020) or a bite from an SFTSV-infected companion animal (Tsuru et al. 2021), SFTSV is usually transmitted through tick bites (Saijo 2019). In the ROK, *Haemaphysalis longicornis*, *Haemaphysalis flava*, *Amblyomma testudinarium*, and *Ixodes nipponensis* are known to vector SFTSV (Yun et al. 2014, 2016). Several studies have suggested that *H. longicornis* comprises the major population of ticks in the ROK (Seo et al. 2021b; Kang et al. 2022) and is considered the main vector and reservoir for SFTSV through transovarial and transstadial transmission (Luo et al. 2015). The prevalence of SFTSV in ticks was determined in various environments using flagging, dragging, or dry ice-baited traps (Seo et al. 2021a, b; Kang et al. 2022). It was also determined in wild animals, including wild deer, goral, raccoon dogs, boars, carrion crows, reptiles, and domestic dogs (Kim et al. 2014; Oh et al. 2016; Suh et al. 2016) in the ROK. Yun et al. (2016) reported SFTSV prevalence in questing ticks in the outbreak areas, including six provinces and two metropolitan cities, based on the number of SFTS cases. Moreover, Kim et al. (2018) detected SFTSV in a patient and the respective tick that bit the patient. However, Chung et al. (2020) could not identify SFTSV from ticks collected by dragging and flagging from surrounding patient’s residence and de-ticking of three companion dogs.

In this study, we describe a molecular epidemiological investigation to verify the association between SFTSV prevalence in ticks around a residence and an SFTS case. This study contributes to the control and understanding of the transmission cycle in ticks that retain SFTSV.

## Methods

### Patient

**Fig. 1:**
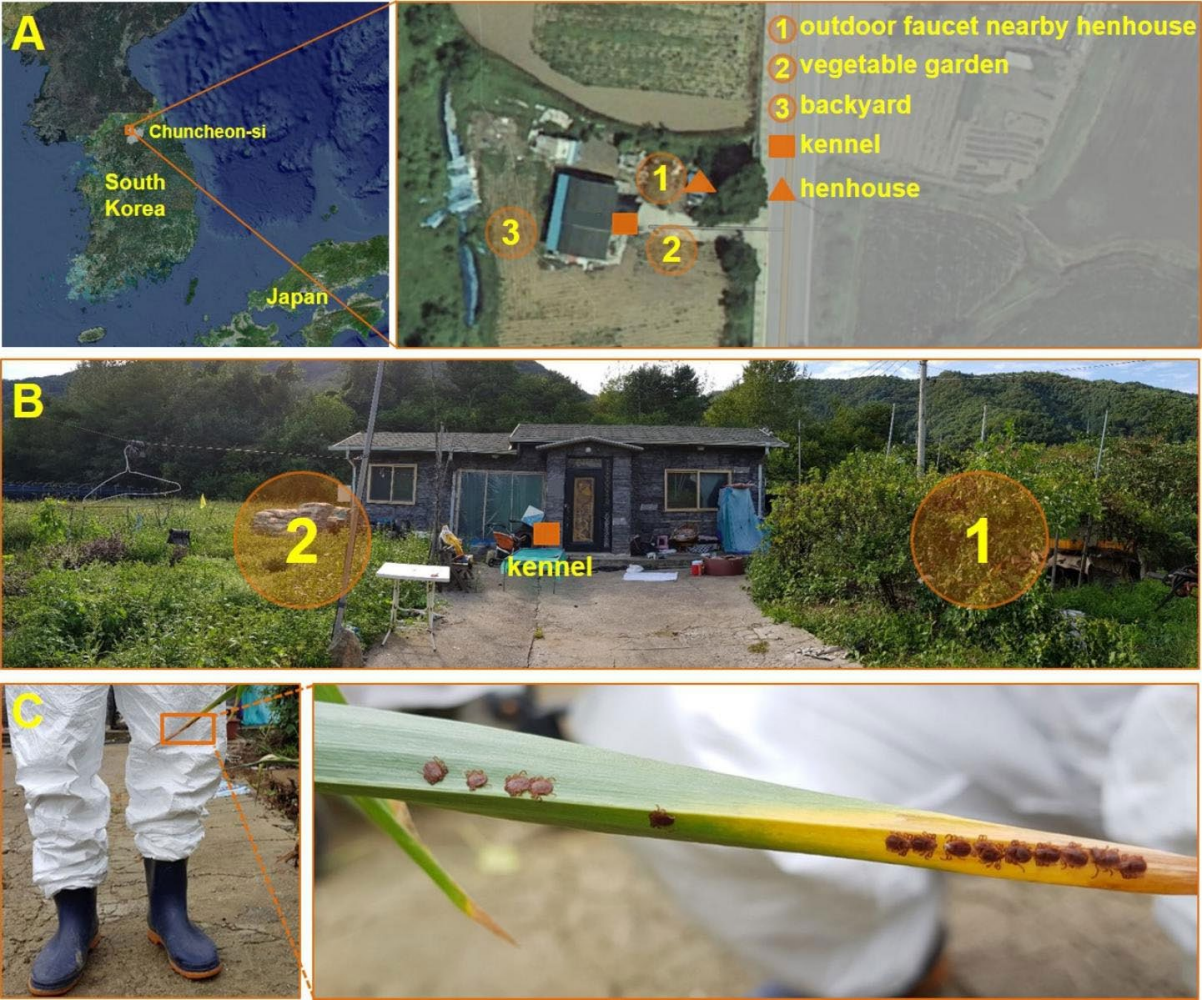
Location of collection site surrounding the house of the patient with SFTS in Chuncheon-si, Gangwon-do, Republic of Korea (A; Image source by Korea Statistical Information Service). An epidemiological investigation was performed in the rural area surrounding the residence of the patient on July 30, 2019. The kennel (■) was outside the house, and the henhouse (▲) was located between the outdoor faucet and some broadleaf trees. (B) The kennel is in front of the house and the outdoor faucet is hidden by trees (1) on the right, and a small vegetable garden (2) on the left. (C) Ticks were identified on a leaf located at the height of the investigator’s knee near the outdoor faucet

The patient was an 82-year-old woman who lived alone in a rural area with a companion dog and grazing chickens around her residence in Chuncheon City, Gangwon Province (ROK) (Fig. 1). The area included a small vegetable garden, kennel, henhouse, and outdoor facet, where the patient spent most of her time working. During the medical history interview, the patient first reported a tick bite on her back on June 19, 2019, a few days after weeding the lawn at her residence without protective equipment. Additionally, the companion dog died in May or June with a large number of ticks attached to its body.

### Collection and identification of tick

Questing ticks were collected by flagging (Ginsberg and Ewing 1989) and dry ice-baited traps (Seo et al. 2021b) at three spots on the vegetation around the house of the patient with SFTS on July 30, 2019 (Fig. 1). These spots were selected based on the activities of the patient in June. After collection, the ticks were placed in a tick collection tube (Seo et al. 2021b) and transported to the laboratory. Species and developmental stages were identified under a dissecting microscope using taxonomic identification keys (Yamaguti et al. 1971). Ticks were placed in cryogenic vials according to species, developmental stage, and collection spots. Each tube contained 1–5 adults or 1–30 nymphs for SFTSV infection assays. They were stored at -70 °C before RNA extraction.

### RNA extraction and one-step RT-PCR

Approximately half of the collected ticks were used to detect SFTSV infection, and tick samples were homogenized in 200 µL of 1× phosphate-buffered saline (PBS) with a Precellys 2 mL Hard Tissue Reinforced ceramic Beads kit (Ck28-R) using a Precellys Evolution homogenizer (Bertin Technologies, Montigny-le-Bretonneux, France). The homogenates were centrifuged at 25,000× ***g*** for 10 min, and 100 µL of the supernatant was used for RNA extraction using a MagMAX *mir*Vana Total RNA Isolation kit (Applied Biosystems, Foster City, CA, USA) with a KingFisher Flex system (Thermo Fisher, Waltham, MA, USA) according to the manufacturers’ instructions. To detect the partial M segment of SFTSV, a reverse transcription polymerase chain reaction (RT-PCR) was conducted using a DiaStar 2X OneStep RT-PCR Pre-Mix kit (SolGent, Daejeon, ROK) with SFTSV-specific MF3 (5’-GATGAGATGGTCCATGCTGATTCT-3’) and MR2 (5’-CTCATG GGGTGGAATGTCCTCAC-3’) primers, which produce 560 bp products (Yun et al. 2014). Reactions were performed with an initial step of 30 min at 50 °C and 15 min at 95 °C for denaturation, followed by 35 cycles of 20 s at 95 °C, 40 s at 58 °C, and 30 s at 72 °C, and a final extension step of 5 min at 72 °C. To avoid cross-contamination, the positive control produced a product of 700 bp as per primers designed by the authors. Amplified PCR products were visualized using 1.5% agarose gel electrophoresis after staining with Safe-Pinky DNA Gel Staining Solution (10,000×) In Water (GenDEPOT, Barker, TX, USA). RNA extraction, RT-PCR amplification, and electrophoresis were performed in separate rooms to minimize contamination.

### Sequencing and phylogenetic analysis

All positive one-step RT-PCR products were sequenced in directions using the RT-PCR primer at Macrogen (Daejeon, ROK), and sequence information was analyzed using Chromas software (v.2.33). The sequences were aligned using CLUSTAL X (v.2.1) and compared with the patient’s SFTSV sequence obtained from the Division of Viral Diseases, Korea Disease Control and Prevention Agency (KDCA) and other SFTSV sequences reported in GenBank (National Center for Biotechnology Information, NCBI, Bethesda, MD, USA). To assess the relationships between SFTSV genotypes, a phylogenetic tree was constructed using the maximum likelihood method based on the Kimura 2-parameter model using MEGA software (v.5.2), and tree stability was assessed using bootstrap analysis with 1,000 replications.

### Statistical analysis

The significance of the different prevalence of SFTSV between each sexual and developmental stage was performed by Fisher’s exact test using Excel 2016 (Microsoft, Redmond, WA, USA). Statistical significance was set at α *=* 0.05.

## Results

### Patient

Two weeks after the tick bite, the patient developed a fever of 37.6 °C with nausea and diarrhea and was admitted to a local clinic on July 3, 2019. No blood-feeding ticks were observed during the physical examination but a tick-bite lesion was confirmed on the patient’s back. Blood samples were collected on admission and tested for SFTSV by the KDCA. The RT-PCR results of the laboratory examination were positive for SFTSV nucleic acid. The patient was transferred to the intensive care unit but died on July 14.

### Tick collections

In total 1,212 ticks were collected from three sites, including 773 females, two males, and 437 nymphs (Table 1). All the questing ticks were morphologically identified as *H. longicornis*. Among the collection spots, the outdoor faucet (83.7%, 1,014 ticks) accounted for more than half of the ticks, followed by the vegetable garden (13.3%, 161 ticks) and backyard (3.1%, 37 ticks). In the sexual and developmental stages, females (63.8%, 773 ticks) were collected more frequently, followed by nymphs (36.1%, 437 ticks) and males (0.2%, 2 ticks). No larval developmental stage ticks were detected.

**Table 1:** Collection of *Haemaphysalis longicornis* from the residence of the human case of SFTS.

Collection spot	Sexual and developmental tick stage																		Total				
	Female						Male						Nymph										
	CT	TT	TP	PP	MIR		CT	TT	TP	PP	MIR		CT	TT	TP	PP	MIR		CT	TT	TP	PP	MIR
Outdoor faucet	591	278	130	20	7.2		2	2	1	0	-		421	140	5	3	2.1		1,014	420	136	23	5.5
Vegetable garden	147	147	30	9	6.1		-	-	-	-	-		14	14	1	0	-		161	161	31	9	5.6
Backyard	35	35	7	1	2.9		-	-	-	-	-		2	2	1	0	-		37	37	8	1	2.7
Total	773	460	167	30	6.5		2	2	1	0	-		437	156	7	3	1.9		1,212	618	175	33	5.3

CT, no. collected ticks; TT, no. tested ticks; TP, no. tested pools; PP, no. positive pools of SFTS virus; MIR, SFTS virus minimum infection rate per 100 ticks (no. positive pools/total no. tested ticks×100)

### Minimum infection rate of SFTSV

Of the 1,212 ticks, 618 ticks were grouped into 175 pools for SFTSV assays. The minimum infection rate (MIR) of SFTSV per 100 ticks (MIR = no. of positive pools/no. of tested ticks×100) was 5.3% (33 pools/618 ticks). Among the collection spots, the vegetable garden (5.6%, 9 pools/161 ticks) had the highest MIR for SFTSV, followed by the outdoor faucet (5.5%, 23 pools/420 ticks) and backyard (2.7%, 1 pool/37 ticks). Based on the sexual and developmental stages, the MIR of SFTSV was higher in adult females (6.5%, 30 pools/460 ticks) than in nymphs (1.9%, 3 pools/156 ticks), whereas no SFTSV-positive pools were observed in adult males. Prevalence of SFTSV did not differ among females, males, and nymphs (Fisher’s exact test: *P* = 0.29).

Of the 33 positive sequences, five representative sequences, without duplicate sequences of each sexual and developmental stage in this study, were selected and deposited in the GenBank database (ON409516-ON409520).

### Phylogenetic analysis

**Fig. 2:**
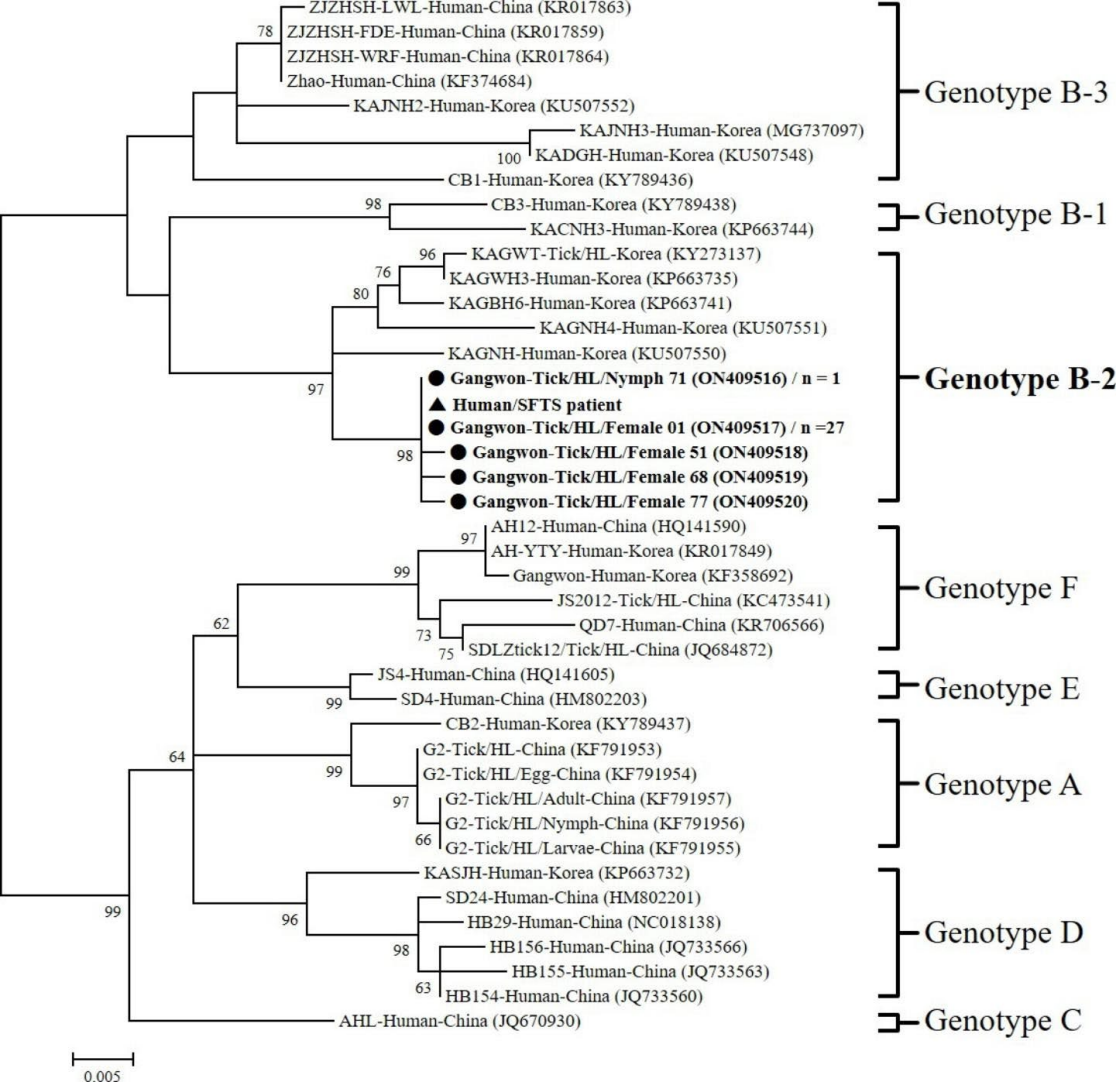
Phylogenetic analysis between SFTSV genotype based on the 548 bp nucleotide sequences of M segments of SFTSV from tick and patient. The numbers on the branches indicate bootstrap percentages based on 1,000 replications. The cut-off value for the consensus tree was 50%. The scale bar represents 0.5% divergence. The dots (**●**) indicate sequences identified in this study, the triangles (**▲**) indicate the patient’s SFTSV sequence. The number (*n*) of sequences with an identical sequence is shown if the sequence was detected in more than one case

Phylogenetic analysis showed that five representative sequences formed a single cluster with the patient’s SFTSV sequence in the B-2 clade with high bootstrap values (Fig. 2). Each of five representative sequences in this study shared 99.6–100% identity. No apparent clustering was observed in the B-2 clade based on the host or developmental stage of the tick. All SFTSV sequences used in this study and their corresponding accession numbers are shown in Fig. 2.

## Discussion

SFTS is a tick-borne contagious viral disease, and *H. longicornis* is the reservoir and vector (Luo et al. 2015). In this study, 1,212 questing ticks of adult and nymphal developmental stages were collected from the residence of a human case of SFTS in the ROK and identified as *H. longicornis*. In the ROK, *H. longicornis* is usually the most dominant species collected from the environment (Chong et al. 2013a, b; Ham et al. 2014; Park et al. 2014; Jung et al. 2019; Kim-Jeon et al. 2019; Seo et al. 2021b; Kang et al. 2022), detached from humans (Yun et al. 2014), and wild animals such as Korean water deer (Kang et al. 2016; Oh et al. 2016), Siberian roe deer, goral, raccoon dogs, Eurasian badger, carrion crows, eagle owl, sparrow hawk (Oh et al. 2016), and domestic pets such as dogs (Kim et al. 2014; Chung et al. 2020). Regarding the seasonal abundance of *H. longicornis* in the ROK, sequential population peaks of nymphs, adults, and larvae are observed from May to June, June to August, and August to September, respectively (Jung et al. 2019; Kim-Jeon et al. 2019). Because in this study we investigated *H. longicornis* in July, more adults (64.0%, 775/1,212) were collected than nymphs (36.1%, 437). In addition, no larvae were collected by any collection method or spot.

SFTSV-positive pools were detected in all collection spots, with an overall MIR of 5.3% per 100 ticks. This result appears to be relatively high compared with other studies, including the detection of SFTSV in Gyeongbuk Province (0.59% MIR, 9 pools/1,529 tested ticks) (Lee et al. 2021) and 14 collection sites in SFTS outbreak areas in the ROK (0.11% MIR, 9 pools/8,313 tested ticks) (Yun et al. 2016). These differences in MIR values may be affected by the collection region, season, survey period, species and developmental stage composition, and the number of ticks tested per pool (Fu et al. 2016).

Based on phylogenetic analysis, SFTSV is systematically classified into six genotypes (A-F) (Fu et al. 2016). Of these, most SFTSV in the ROK is genotype B, which is divided into three strains (B-1, B-2, and B-3) (Yun et al. 2020). In this study, phylogenetic analysis of the five representative sequences of SFTSV based on the partial M segment was clustered into genotype B-2. Interestingly, nos. 71 and 01 (accession nos. ON409516 and ON409517) representing 28 SFTSV-positive sequences in ticks, showed 100% identity with the patient’s virus sequence. Additionally, nos. 51, 68, and 77 (accession nos. ON409518-ON409520) showed a difference between two nucleotide bases with other sequences in the same clade. These two differences may be caused by replication mutations or sequencing errors (Fu et al. 2016).

Since the first report on the molecular detection of the SFTSV in ticks (Yun et al. 2014), numerous studies on SFTSV infection in ticks have been performed in the ROK. However, no reported cases have detected the presence of identical SFTSV in patient as well as questing ticks, particularly those collected from the patient’s house. The presented results strongly support the link between SFTSV-infected ticks and the patient with SFTS with a history of tick bites. Some studies have identified SFTS pathogens in engorged ticks from patients with SFTS in the ROK and China (Wang et al. 2014; Kim et al. 2018; Tong et al. 2020). However, to the best of our knowledge, this is the first case to provide molecular evidence with a high match (99.6–100%) between SFTSV from a patient and questing ticks in the same living environment. However, this study has a limitation considering direct SFTSV transmission from ticks to humans because we did not test engorged tick specimens from the patient with SFTS. To better understand the potential risk sources of SFTSV from ticks to humans, further studies should be conducted to investigate the pathogens in ticks that bite humans to determine whether the pathogens found in questing ticks are the same as those found in engorged ticks from SFTS cases.

## Conclusion

*Haemaphysalis longicornis* collected from an SFTSV-confirmed patient’s residence were infected with the virus. The ticks had a high infection rate (5.3% MIR). Phylogenetic analysis indicated a 99.6–100% match between the M segment sequences of SFTSV from the ticks and the SFTSV from our patient. To our knowledge, this is the first report to identify the SFTSV from ticks collected from a residence of a patient with SFTS in the ROK. These results may help establish risk assessments for patients with SFTS and control policies against tick that transmit SFTS.

## Data Availability

The datasets analyzed during the current study are available from the corresponding author on reasonable request.
